# The Urine, the Common Poisons, and the Milk

**Published:** 1890-01

**Authors:** 


**The Urine, The Common Poisons, and The Milk.** Memoranda, Chemical and Microscopical, for Laboratory Use. By J. W. HOLLAND, M. D., Professor of Medical Chemistry and Toxicology, Jefferson Medical College, of Philadelphia. Illustrated. Third edition, revised and much enlarged. Philadelphia: P. Blakiston, Son & Co., 1012 Walnut Street. 1889.

Prof. Holland has made a useful guide for the laboratory student in this neat book of eighty-four pages, printed on one side of each leaf. It also becomes a useful book of working reference for a physician who must needs make his own urinary examinations and do sundry other work in minor chemistry and toxicology. The addition of instructions in milk examinations makes it of further value for health officers in the smaller cities and towns that do not have regularly appointed official chemists.

